# Unilateral Facial Swelling as the Inaugural Sign of Anti-NPX2 Dermatomyositis: A Diagnostic Challenge in Oral Medicine

**DOI:** 10.1155/crid/3952741

**Published:** 2025-12-01

**Authors:** Françoise Tilotta, Hadrien Le Vayer, Olivier Bory, Caroline Morbieu, Loredana Radoi

**Affiliations:** ^1^Inserm UMR_S 1333 Oral Health, Université Paris Cité, Montrouge, France; ^2^Department of Oral Medicine, AP-HP Paris Saclay-Sainte-Périne Hospital, Paris, France; ^3^Faculty of Health, Université Paris Cité, Montrouge, France; ^4^Department of Oral Medicine, AP-HP Paris Nord-Louis-Mourier Hospital, Colombes, France; ^5^Coordinated Department of Internal Medicine, AP-HP Paris Nord-Louis-Mourier Hospital, Colombes, France; ^6^Inserm ECEVE, Université Paris Cité, Paris, France; ^7^Inserm CESP, Université Paris Cité, Villejuif, France

**Keywords:** autoimmune disease, dermatomyositis, facial swelling, inaugural, oral manifestations, periorbital edema

## Abstract

A 41-year-old female patient in apparent good health presented to the dental emergency department with a 6-week history of painless, nonpruritic left facial swelling, and dotted with purplish patches. She had consulted different physicians and dentists and received several ineffective treatments. After ruling out an oral cause, standard blood tests were initially prescribed. They showed an isolated elevation of creatine phosphokinase (CPK). Muscular weakness of the left arm and leg, pain on mobilization of the left thigh, and erythematous plaques on the left body then appeared. Infectious, granulomatous, and autoimmune diseases were investigated. Magnetic resonance imaging (MRI) showed diffuse muscle infiltration, suggestive of myositis. Myositis-specific dot immunoassay identified antinuclear matrix protein 2 (NXP2) autoantibodies, enabling the diagnosis of dermatomyositis. The inaugural orofacial manifestations of dermatomyositis can be confused with pathologies of dental cause, such as cellulitis. In the absence of local warning signs, oral health professionals must consider a systemic etiology and rapidly refer the patient to avoid patient wandering.

## 1. Introduction

Autoimmune diseases comprise a broad group of chronic inflammatory disorders triggered by the loss of immunological tolerance to the body's own components. Overall, autoimmune diseases affect 4%–5% of the general population in industrialized countries, with a significantly higher prevalence in women [1].

Orofacial manifestations frequently occur in autoimmune diseases (e.g., vasculitis, scleroderma, systemic lupus erythematosus, sarcoidosis, inflammatory myopathies, rheumatoid arthritis, and Sjögren's syndrome), especially at early stages [2]. These manifestations are generally not pathognomonic but can be the first signs of the disease or its only identifiable manifestations [3]. They include orofacial swelling or pain, temporomandibular joint dysfunction, dysphagia or muscular dystonia, hyposalivation, burning mouth syndrome, and various cutaneous and/or mucosal lesions (e.g., candidiasis, cheilitis, erythematous patches, ulcers, and gingival enlargements) [3].

Identifying these manifestations is essential, as it allows for early diagnostic investigation and timely referral to a general practitioner or specialist for appropriate management. These manifestations pose a challenge for oral healthcare practitioners, as they can be easily mistaken for local or regional orofacial conditions, potentially leading to misdiagnosis and inappropriate treatment that may worsen the patient's overall health status. Oral health professionals should consider a systemic disease when encountering any atypical orofacial manifestation, after ruling out local causes (e.g., dental, periodontal, salivary, or temporomandibular diseases). Among these systemic diseases, anti-NXP2 antibody-related dermatomyositis (DM) with orofacial involvement is extremely rare. We report an atypical case in which the oral manifestations were inaugural and unilateral, resulting in a diagnostic and therapeutic delay of several weeks.

## 2. Case Presentation

A 41-year-old female patient presented at the emergency consultation of a hospital-based oral medicine service in the Paris region, referred by the general emergency department for a left-sided facial swelling persisting for about 1 month, associated with unilateral pulsatile headaches on the left side.

Her medical history included endometriosis, uterine fibroid, iron deficiency anemia due to an ulcerated peptic esophagitis, and *Helicobacter pylori* gastritis. At the time of consultation, she was on progestin-based oral contraception and pantoprazole.

The patient had previously been seen in general medicine, ophthalmology, and three times in general emergency care. The various consulted physicians suspected an infectious cause (facial cellulitis) and prescribed several antibiotic treatments (cefuroxime for 7 days, pristinamycin for 10 days, and then amoxicillin–clavulanic acid for 7 days) without significant improvement. A 7-day course of prednisolone was also prescribed by the emergency physician, but without effect. Blood tests (complete blood count, ferritin, liver and kidney function evaluation, inflammatory markers, immunoglobulins E from multiple allergens, ionogram, fasting glucose, and protein electrophoresis) were normal except for an elevated C-reactive protein (CRP) (32 mg/L).

The patient reported that the facial swelling was progressively accompanied by the appearance of reddish-purple patches initially under the orbit, spreading to the lower border and angle of the mandible. A recent craniofacial computed tomography (CT) scan had revealed a periapical radiolucency on the upper right central incisor, leading emergency physicians to refer the patient for a dental evaluation.

Clinical examination showed an afebrile but asthenic patient. She denied joint pain, weight loss, or appetite loss but reported mild dysphagia for solid aliments. There were no recent changes in diet, cosmetic products, or toothpaste use. Outside of the face, the exposed skin, scalp, and nails showed no abnormalities.

**Figure 1:**
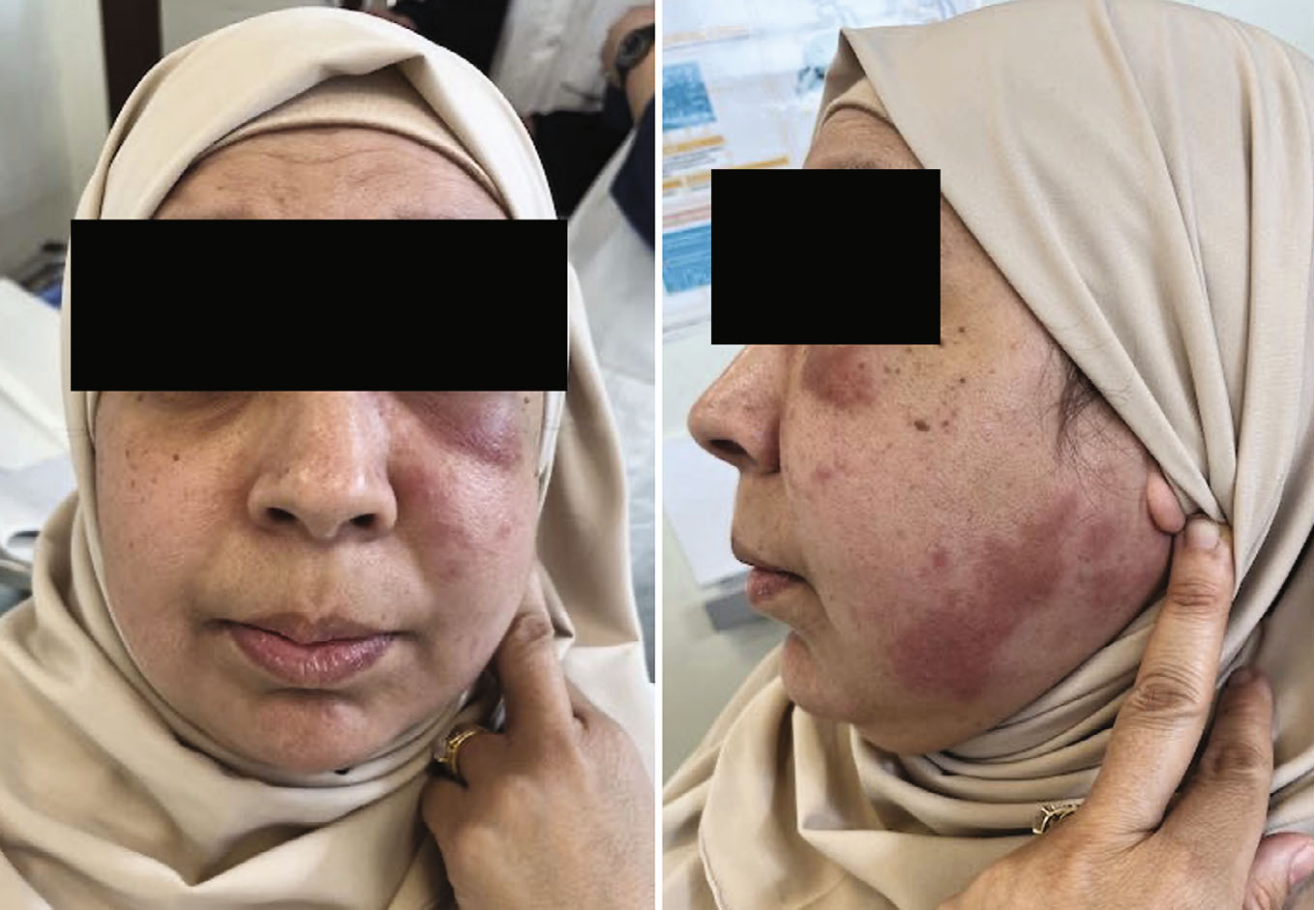
Front and profile photos: swelling of the middle and lower regions of the left hemiface, accompanied by multiple reddish-purple plaques.

The extraoral examination revealed swelling of the mid and lower left hemiface, without frontal involvement. The swelling was not painful or itchy and was interspersed with reddish-purple plaques in the infraorbital and cheek areas, extending to the lower border and angle of the mandible. The lower eyelid was swollen and purplish (Figure 1). Palpation was painless, without subcutaneous crepitus. No cervical–facial lymphadenopathy was detected.

**Figure 2:**
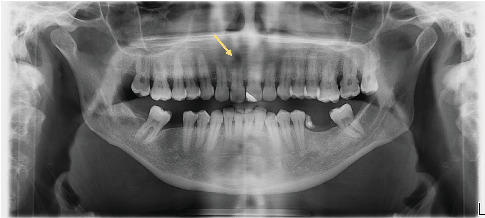
Panoramic X-ray: radiolucent periapical image on the right maxillary central incisor (yellow arrow) and the absence of any notable abnormalities on the left side that could explain the clinical presentation.

The intraoral examination was normal; the clinical tests performed on the tooth suspected to be the cause by the previously consulted practitioners (right maxillary central incisor) showed that it was asymptomatic and, therefore, could not be responsible for the clinical presentation. The absence of any other dental, bone, or salivary abnormalities, aside from moderate periodontal disease, ruled out an oral etiology for the facial swelling (Figure 2).

Given the clinical presentation (headache, unilateral facial swelling, and reddish-purple plaques) and the absence of a dental cause, the possibility of a systemic disease with initial facial manifestations was considered, including lupus erythematosus, angioedema, vasculitis, or DM.

A specialist consultation (internist and dermatologist) was scheduled, and further laboratory tests were prescribed, including a complete blood count, thyroid-stimulating hormone (TSH), serum protein electrophoresis, autoimmune panel (antinuclear antibodies and anti-DNA antibodies), creatine phosphokinase (CPK), and viral serologies (human immunodeficiency virus (HIV), hepatitis C virus (HCV), hepatitis B virus (HBV), and Epstein–Barr virus (EBV)).

The results were normal except for elevated CPK levels at 593 U/L, 9 days after the emergency consultation in the oral medicine service (normal range: 10–200 U/L).

**Figure 3:**
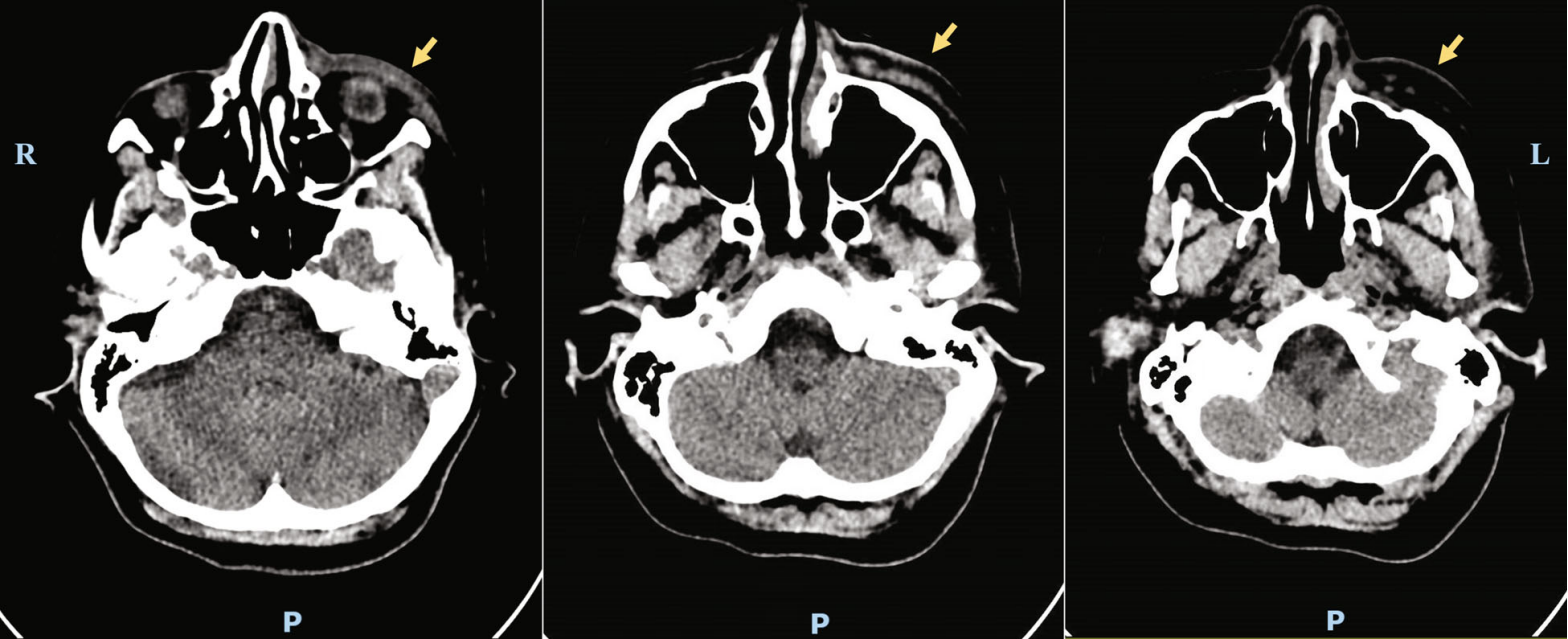
Contrast-enhanced brain angio-CT: normal aspect except for infiltration and thickening of the soft tissues of the left hemiface (yellow arrow).

Within a week after the oral consultation, the patient developed progressive muscle weakness in both upper and lower limbs associated with paresthesia in the left hand and foot, but without motor deficit. She also reported pain in the left hip. These symptoms, emerging approximately 5–6 weeks after the onset of facial swelling, prompted hospitalization in the internal medicine department for further investigation. Subsequent blood tests revealed a marked elevation of CPK to 5019 U/L, measured within the 1-month interval between the emergency oral medicine consultation and admission to the internal medicine department. A contrast-enhanced brain angio-CT was performed to rule out cerebral thrombophlebitis but it was normal. It showed only infiltration and thickening of the soft tissues of the left hemiface (Figure 3).

The clinical course progressed with the appearance of erythematous plaques on the posterior–lateral left thigh and the upper-lateral quadrant of the right breast. The left thigh was painful upon movement.

New diagnostic hypotheses were explored, including infectious (syphilis, Lyme disease, Chagas disease, bartonellosis, trichinellosis, invasive fungal infection, etc.), granulomatous (sarcoidosis), and autoimmune (DM) etiologies.

**Figure 4:**
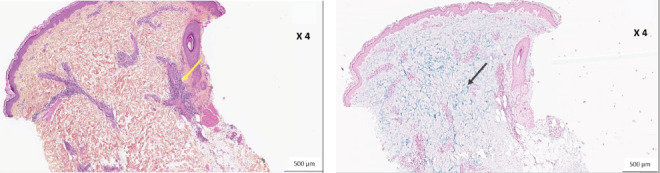
Histological sections on paraffin-embedded samples of skin biopsy from the thigh. (a) Hematoxylin–eosin–safran (HES) staining showing a perivascular lymphocytic infiltrate (yellow arrow) in the dermis—magnification 4x. (b) Alcyan blue staining showing mucin deposition (in blue, black arrow)—magnification 4x.

A skin biopsy from the thigh showed a lymphocytic infiltrate and mucin deposition in the dermis (Figure 4).

Magnetic resonance imaging (MRI) of the pelvis and left psoas revealed diffuse and symmetric increased T2-weighted signal suggestive of an inflammatory myopathy. A myositis-specific antibody panel (commercial dot immunoassay) identified antinuclear matrix protein 2 (NXP2) autoantibodies. As investigations continued, a new skin lesion appeared on the left arm, accompanied by motor deficits. The diagnosis of anti-NXP2 DM, complicated by motor deficits in the left upper and lower limbs, was confirmed. Thoraco-abdomino-pelvic CT scan showed no pulmonary involvement.

Initial treatment with intravenous methylprednisolone (1 g over 3 days) followed by oral prednisolone (80 mg/day) and methotrexate (25 mg/week subcutaneous) led to temporary clinical improvement, until relapse of pain and motor deficit in the left upper and lower limbs, requiring the administration of intravenous immunoglobulins (Tegeline 2 g/kg body weight every month). Dermocorticoids were applied to the skin lesions. Four-limb physical therapy was also prescribed. Clinical and biological control of the disease was achieved after 12 months, allowing treatment doses reduction to 6 mg/day of prednisolone and 10 mg/week of methotrexate.

## 3. Discussion

We report a rare case of DM with inaugural facial signs and unilateral involvement that led to the patient's misdiagnosis and wandering. Diagnosis was a challenge for both oral care practitioners and physicians.

DM is a rare autoimmune disease among idiopathic inflammatory myopathies [4]. It manifests as erythematous skin plaques, along with muscle weakness and pain [5]. The muscular and cutaneous phenotypes of different types of DM vary depending on the type of autoantibodies present (Table 1). The clinical presentation can be severe, with numerous complications (pulmonary, cardiac, cancer-related, etc.) [6], potentially leading to the patient's death [7].

The global incidence of inflammatory myopathies in the general population ranges from 1.16 to 19 per 1,000,000 new cases per year. Their prevalence is estimated to be between 2.4 and 33.8 per 100,000 inhabitants [14]. The frequency of inflammatory myopathies increased over time, which may reflect progress in diagnostic performance, although there is still a need to increase the level of awareness with regard to these diseases, as attested by its considerably delayed diagnosis.

DM affects women twice as often as men and can occur at any age, with two peak incidence periods: between 5 and 14 years old (juvenile DM) and between 50 and 60 years old [5].

The diagnosis of DM is guided by the presence of characteristic skin signs, often the first manifestation, affecting exposed areas such as the face, along with bilateral and symmetrical muscle weakness. It is confirmed by elevated muscle enzymes, skin or muscle biopsy, and the presence of myositis-specific autoantibodies. Muscle MRI further confirms muscle involvement.

In this patient, periorbital violaceous (heliotrope) erythema with edema characteristic of anti-NXP2 antibody DM was observed. However, it is important to note that these manifestations were unilateral in this case, whereas the literature primarily reports bilateral and symmetrical facial edema associated with DM [15]. Anti-NXP2 DM rarely manifests unilaterally in the orofacial region, and initial facial swelling can easily mimic a dental infection. This highlights the importance for oral health professionals to recognize systemic autoimmune etiologies as a cause of atypical orofacial swelling. We found in the literature a single article similar to ours, by Saleh and Saleh, reporting a case of anti-NXP2 antibody positive DM presenting as unilateral periorbital heliotrope rash [16]. Compared with the case described by Saleh et al., our patient presented at a younger age and initially with unilateral facial swelling rather than a heliotrope rash. The interval between the onset of symptoms and diagnosis was longer in our patient than in the case reported by Saleh et al., and the CPK elevation was more progressive and more extensive. A 1-month history of muscle weakness was noted at initial presentation in the case of Saleh et al., whereas no such symptom was observed in our patient, whose muscle involvement was unilateral and appeared later than the facial manifestations. Contrary to the case of Saleh et al., neither Gottron's papules nor calcinosis cutis were reported, but the patient reported mild dysphagia. The other skin lesions (on the left thigh and left arm) as well as the motor deficits were unilateral as was the facial swelling, which represents an atypical form of the disease in our patient. Initially unilateral, these signs became bilateral and severe as the disease progressed. MRI revealed diffuse muscle infiltration of the pelvis and psoas muscles, in both cases. These differences illustrate the variability of anti-NXP2 DM with unilateral orofacial onset.

Anti-NXP2 antibodies are specific to myositis, just like anti-Mi2, anti-SAE, anti-TIF-*γ*, and anti-MDA5 antibodies, and are not observed in other autoimmune diseases. Since 2018, these five antibodies have been considered diagnostic criteria for DM by the European Neuromuscular Center (ENMC) [4].

Anti-NXP2 antibodies are identified in 14%–25% of DM cases in Caucasian individuals [17]. However, the incidence of anti-NPX2 antibody-related DM presenting in the orofacial region is extremely rare and not numerically defined in current literature. Only isolated case reports describe facial or periorbital swelling, usually bilateral, as an early manifestation of the disease. Anti-NXP2 antibodies are associated with recurrent cutaneous forms in both children and adults, and with a higher incidence of cancer in adults, particularly in males [8]. In this patient, a paraneoplastic DM was investigated and ruled out after mammography, positron emission tomography (PET) scan, and colonoscopy.

The cutaneous manifestations of DM may be the initial signs of the disease, with muscle involvement appearing only a few weeks or months later, as in our patient. The characteristic features are heliotrope periorbital edema, Gottron's sign and Gottron's papules (erythema and hyperkeratosic red papules on the back of the metacarpophalangeal and interphalangeal joints), periungueal telangiectasia and violaceous erythema often associated with mild edema usually located on the face, neck, shoulders, or hands [18].

The cutaneous manifestations of DM can be atypical and include blisters, ulcerations, Raynaud's phenomenon, and calcinosis. Dwivedi et al. reported a case of juvenile DM with bilateral periorbital edema without muscle weakness, associated with elevated transaminases [7], with a fatal outcome. Facial and laryngeal edema are also described as initial manifestations, sometimes accompanied by dyspnea and acute respiratory failure [6]. Bilateral facial ulcerations have also been reported in association with bilateral purple facial erythema in a case of anti-MDA5 DM.

Initial isolated bilateral facial edema may occur, especially in amyopathic forms which delay diagnosis and can lead to severe clinical presentations of the disease [6]. Muscular involvement is frequent, but amyopathic forms are described, especially in anti-MDA5 DM. Extra-musculo-cutaneous features of DM, including interstitial lung disease, arthritis and constitutional signs, such as fever, are particularly frequent in anti-MDA5 DM.

The differential diagnosis for unilateral facial swelling includes angioedema, vasculitis, sarcoidosis, and lymphoproliferative disorders such as lymphoma [19]. Angioedema presents as a sudden, soft, nonpainful swelling, often triggered by an allergic reaction or medication such as angiotensin-converting enzyme inhibitors. Vasculitis causes a painful, inflammatory swelling with purpura or other systemic signs. Finally, lymphoma produces a firm, painless, and progressive swelling, often accompanied by cervical lymphadenopathy and sometimes by superior vena cava syndrome.

In our case, the absence of pain, fever, or lymphadenopathy, along with elevated muscle enzymes and MRI evidence of myositis, helped to exclude these conditions.

The management of patients with DM is carried out in a specialized center in collaboration with the primary care physician. The mainstay of treatment consists of corticosteroid therapy, usually combined with methotrexate [20]. Other immunosuppressive treatments, as well as therapy with rituximab or intravenous immunoglobulins, may also be initiated depending on the clinical manifestations and associated complications [21]. Physical therapy is an integral part of patient management.

In the reported case, intravenous pulse corticosteroid therapy followed by oral administration, associated with subcutaneous methotrexate and intravenous immunoglobulin, led to a significant reduction in skin lesions and motor deficits, as well as the normalization of CPK levels after 12 months of treatment.

Orofacial manifestations can easily be mistaken for dental pathologies such as cellulitis, leading to inappropriate treatments by oral health specialists and diagnostic delays. When faced with orofacial swelling, they must be able to rule out local causes and, in the absence of local warning signs, consider a systemic etiology. Prompt referral to the appropriate specialist is crucial to ensure proper management of the condition and to prevent severe complications, including potential fatal outcomes.

## Figures and Tables

**Table 1:** DM phenotypes according to myositis-specific autoantibodies.

**Present autoantibodies**	**Cutaneous involvement**	**Muscular involvement**	**Extra-cutaneous and extra-muscular involvement**	**Association with cancer**
Anti-NXP2	Calcinosis/subcutaneous edema/periorbital rash/photosensitivity/« mechanic's hands »	Severe involvement/vasculopathy and edema/myalgia/dysphagia	Rare	Moderate but more signifiant in men [8]
Anti-MDA5	Severe skin ulcers, sometimes/palmar papules	Moderate involvement with normal CPK levels/frequent « amyopathic » form	Severe and progressive ILD in 80% of cases, potentially life-threatening [9]/constitutional fever/inflammatory joint involvement with arthritis and arthralgia	No
Anti-SAE	Typical and severe involvement/occasionally purple rash	Dysphagia/moderate involvement with normal or slightly elevated CPK levels	Fever/deterioration of general condition	Moderate [10]
Anti-Mi2	Typical erythema in photo-exposed areas/lilac erythema of the eyelids/Gottron's papules [11]	Severe involvement with high CPK levels and significant inflammation	Rare	Low [11]
Anti-TIF1-gamma	Severe and recurrent involvement/hyperkeratotic Gottron's papules/psoriasiform lesions/patch « red or white » [12]	Moderate involvement with low CPK levels/frequent « amyopathic » form	Rare	High [13]

*Note:* Patch “red or white”: hypopigmented or telangiectatic lesions. “Mechanic's hands”: fissured and pigmented hyperkeratosis on the pads and lateral surfaces of the fingers.

Abbreviations: Anti-NXP2, antinuclear matrix protein 2 autoantibodies; Anti-MDA5, antimelanoma differentiation-associated protein 5 autoantibodies; Anti-Mi2, anticomplex nucleosome remodeling histone deacetylase autoantibodies; Anti-SAE, antismall ubiquitin-like Modifier-1 activating enzyme autoantibodies; Anti-TIF1-gamma, antitranscription intermediary factor 1-gamma autoantibodies; ILD, interstitial lung disease.

## Data Availability

Data sharing is not applicable to this article as no datasets were generated or analyzed during the current study.
